# Supplementary material for: Impact of Ed-LinQ: A Public Policy Strategy to Facilitate Engagement between Schools and the Mental Health Care System in Queensland, Australia
Source: Int J Environ Res Public Health. 2021 Jul 27;18(15):7924. doi: 10.3390/ijerph18157924 (PMC8345643; doi:10.3390/ijerph18157924)
Supplement: Supplementary file 1 [file ijerph-18-07924-s001.zip › Supplementary File 1.pdf]

Survey Monkey online survey

Ed-LinQ Initiative Impact Assessment Survey

***1. Please provide the postcode for the school**

ZIP/Postal Code:

2. Please provide your name and contact number.

This question is OPTIONAL.

These details will be used if we need to verify responses.

Name:

Phone Number:

***3. Please enter your role title (e.g. Guidance Officer, Counsellor, Principal, School Nurse, Teacher, Chaplain)**

***4. Please provde details of the number of students that are enrolled at your school by age group.**

0 - 5 years

6 - 9 years

10 - 14 years

15 - 18 years

***5. Please select the sector applicable to your school.**

Government Independent Catholic

Ed-LinQ Initiative Impact Assessment Survey

***6. Do you know about the Queensland Ed-LinQ Initiative?**

- Yes
- No

Ed-LinQ Initiative Impact Assessment Survey

***7. Is your school currently undertaking other mental health initiatives?**

- Yes
- No

If Yes, please list them:

***8. Would your school like to undertake other mental health initiatives?**

- Yes
- No

If Yes, please list them:

Ed-LinQ Initiative Impact Assessment Survey

***9. Has the Queensland Ed-LinQ Initiative been presented at your school?**

Yes

No

10. If you answered Yes to question 7, please answer the following:

Who presented the Initiative?

When? (Date approximately)

11. If you answered No to question 7, please answer the following:

How do you know about the Ed-LinQ Initiative?

***12. Has your school discussed the possibility of engaging in the Queensland Ed-LinQ Initiative?**

Yes

No

***13. Is the Queensland Ed-LinQ Initiative part of your school's official documents, procedures, plans and reports?**

Yes

No

If Yes, in which documents is it included?

***14. Has your school actually implemented any aspects of the Ed-LinQ program?**

Yes

No

15. If you answered Yes to question 12, please indicate the extent of the implementation

Pilot implementation

Routine program

Ed-LinQ Initiative Impact Assessment Survey

***16. Please indicate the Ed-LinQ related initiatives that have/are occurring in your school?**

	Yes	No
Inclusion in school policy and protocols	◯	◯
Distribution of information on available services for students	◯	◯
Training of student welfare staff (e.g. Guidance, Counselling, Nursing et al)	◯	◯
Training of senior administration staff (Principal, Head of Middle School etc.)	◯	◯
Training of general teaching staff	◯	◯
Assessment/early identification of the student with mental health problems	◯	◯
Defined referral processes to Child and Youth Mental Health Services or other services (e.g. headspace)	◯	◯
Case coordination/monitoring/follow up with the students	◯	◯
Case coordination/monitoring/follow up with the mental health/primary health care professionals	◯	◯
Provision of treatment at the school for students with mental health related problems	◯	◯
Introduction of new social and emotional learning programs or modification of learning programs for all students	◯	◯
Introduction of new social and emotional learning programs or modification of learning programs for students with learning difficulties, conduct issues or those with a mental health problem	◯	◯

***17. Has your school incorporated indicators or measures related to Ed-LinQ into its routine monitoring system, e.g.number of referrals to primary health care providers (e.g. GPs or psychologists), number of cases of students with a diagnosed mental illness, etc.?**

◯ Yes ◯ No ◯ Not sure

If Yes, please list those indicators your school is using

***18. Please indicate the activities related to the social and emotional wellbeing that your school is undertaking and the extent of implementation?**

"Mandatory" means all students are required to take this activity, while "Elective" means students may or may not undertaken this activity.

	Mandatory	Elective	Not Sure	Not Applicable
General pastoral care	○	○	○	○
Personal development	○	○	○	○
Positive psychology	○	○	○	○
Resilience training and development	○	○	○	○
Mental Health (e.g. Kids matter, Mind Matters)	○	○	○	○
Alcohol and drug education	○	○	○	○
Sexuality education	○	○	○	○

Other? Please list or describe

***19. To what extent has the participation of your school in the Ed-LinQ Initiative contributed to**

	Extremely	Substantially	Moderately	Slightly	Not At All	Not Sure
Improved interagency communication with Child and Youth Mental Health Services, Primary Health Care providers etc.?	○	○	○	○	○	○
Improved knowledge among school staff of the mental health support needs of young people?	○	○	○	○	○	○
Improved student access to mental health assessment and early intervention?	○	○	○	○	○	○
Improved school staff access to mental health resources and guidance?	○	○	○	○	○	○
Increased capacity of school counselors and other student welfare personnel to support students who have, or are at risk of developing mental illness?	○	○	○	○	○	○
Improved coordination across the various school mental health and wellbeing programs such as KidsMatter, MindMatters, Positive Psychology, etc.?	○	○	○	○	○	○
Improved student attendance?	○	○	○	○	○	○
Improved student academic performance?	○	○	○	○	○	○
Improved management and/or successful intervention for students with a mental health problem?	○	○	○	○	○	○

20. Please provide an example/examples of improvements resulting from your participation in the Ed-LinQ Initiative in the following categories, where applicable?

Improved interagency communication (with Child and Youth Mental Health Services, Primary Healthcare providers etc.)

Improved knowledge among school staff of the mental health support needs of young people

Improved student access to mental health assessment and early intervention

Improved school staff access to mental health resources and guidance

Increased capacity of school counselors and other student welfare personnel to support students who have, or are at risk of developing mental illness

Improved coordination across the various school mental health and wellbeing programs (such as KidsMatter, MindMatters, Positive Psychology, etc.)

Improved student attendance

Improved student academic performance

Improved management and/or successful intervention for students with a mental health problem

***21. Overall, what is your degree of satisfaction with the Ed-LinQ Initiative?**

- Very dissatisfied
- Dissatisfied
- Neutral
- Somewhat satisfied
- Very satisfied

***22. Is your school currently undertaking other mental health initiatives?**

- Yes
- No
- Not Sure

If Yes, please list them:

***23. Are there other initiatives related to mental health that your school would like to undertake?**

- Yes
- No
- Not Sure

If Yes, please list them:

Ed-LinQ Initiative Impact Assessment Survey

24. Do you have any other comments on the Ed-LinQ initiative including any changes your would recommend?

Ed-LinQ Initiative Impact Assessment Survey

If you have any questions regarding this survey, please contact Marion Wands at ConNetica Consulting on 07 5491 5456 or by email at mwands@connetica.com.au .

Thank you for your participation and contribution to this Ed-LinQ evaluation.
