# Supplementary material for: Impact of Ed-LinQ: A Public Policy Strategy to Facilitate Engagement between Schools and the Mental Health Care System in Queensland, Australia
Source: Int J Environ Res Public Health. 2021 Jul 27;18(15):7924. doi: 10.3390/ijerph18157924 (PMC8345643; doi:10.3390/ijerph18157924)
Supplement: Supplementary file 1 [file ijerph-18-07924-s001.zip › Supplementary File 2.pdf]

Child and Youth Providers of

***1. Please select your work location from the list provided.**

If your location is not listed, please select Other.

- Caboolture
- Cairns
- Children's Health Queensland Hospital and Health Service
- Darling Downs
- Gold Coast
- Mackay
- Metro South
- Sunshine Coast
- Townsville
- West Moreton
- Other

Child and Youth Providers of

***2. Your selection "Other" in the previous question.**

Please enter the name of your work location in the text box provided.

3. Please provide your name and contact number.

This question is OPTIONAL.

These details will be used if we need to verify responses.

Name:

Phone Number:

4. What program do you work in?

- CYMHS
- headspace
- Evolve
- Other mental health or health related support service

***5. Please enter your current role (e.g. Director, Team Leader, Intake and Assessment)**

***6. Please indicate the number of years that you have worked in CYMHS/Evolve/headspace/other mental health and health related support services role.**

- Less than 12 months
- 1 - 2 years
- 3 - 5 years
- 6 - 10 years
- More than 10 years

***7. Please indicate the number of staff in your team at your location.**

- 0 - 3 staff
- 4 - 6 staff
- 7 - 10 staff
- More than 10 staff

Child and Youth Providers of

***8. Do you know about the Queensland Ed-LinQ Initiative?**

- ◯ Yes
- ◯ No

***9. Are there other initiatives related to school-based mental health and/or suicide prevention that are occurring in your area/District where your organisation is currently involved?**

Yes No Not sure

If Yes, please list them:

***10. Do you believe more needs to be done to address the mental health needs of school-age children and young people?**

Yes No

If Yes, what do you think are the priorities for action? Please list them.

Child and Youth Providers of

***11. Please rate your experiences and views of the Ed-LinQ Initiative, on a scale from 1 - Strongly agree to 5 - Strongly disagree or indicate where you don't know.**

Please note that each question requires an answer.

	1. Strongly Agree	2. Agree	3. Neither agree nor disagree	4. Disagree	5. Strongly disagree	Don't know
I have enough information about the Ed-LinQ Initiative.						
I know and understand what the Ed-LinQ Initiative is aiming to achieve.						
I have enough information about the Ed-LinQ Initiative to know what I can do to support the initiative.						
I know and understand the role of the Ed-LinQ Coordinator.						
When the Ed-LinQ Initiative was introduced under the Queensland Mental Health Plan 2007-2017, there was sufficient consultation with CYMHS staff.						
The Ed-LinQ Initiative is well-supported by the evidence on child and youth mental health.						
The documentation supporting the Ed-LinQ Initiative is adequate.						
There has been sufficient State-wide support for the Ed-LinQ Initiative.						
There has been sufficient support for the Ed-LinQ Initiative in my District or Health and Hospital Service (HHS).						
There is sufficient flexibility in the Ed-LinQ Initiative Framework to account for local needs.						
The investment of time and funds in Ed-LinQ is appropriate given it's objectives.						
The cost of Ed-LinQ is justified given the results to date.						

Child and Youth Providers of

***12. Please rate your experiences and views about Ed-LinQ as an initiative driven by CYMHS, on a scale from 1 - Strongly agree to 5 - Strongly disagree or indicate where you don't know.**

Please note that each question requires an answer.

	1. Strongly Agree	2. Agree	3. Neither agree nor disagree	4. Disagree	5. Strongly disagree	Don't know
The Ed-LinQ Initiative receives visible support from the CYMHS leadership	◯	◯	◯	◯	◯	◯
The Ed-LinQ Initiative is well regarded by Senior CYMHS staff in this District / Hospital and Health Service (HHS).	◯	◯	◯	◯	◯	◯
Ed-LinQ is well aligned with the priorities of CYMHS in this District or HHS.	◯	◯	◯	◯	◯	◯
The Ed-LinQ Initiative is reflected in the business plans and resource allocations for this District / HHS.	◯	◯	◯	◯	◯	◯
CYMHS business systems and policies support the effective operation of Ed-LinQ.	◯	◯	◯	◯	◯	◯
Schools are an important partner in providing effective child and youth mental health services in this District / HHS.	◯	◯	◯	◯	◯	◯
It is important for CYMHS to have a presence in schools.	◯	◯	◯	◯	◯	◯
The Ed-LinQ Initiative has facilitated better interactions with school authorities in this District or HHS.	◯	◯	◯	◯	◯	◯

*13. Has the Queensland Ed-LinQ Initiative resulted in changes to CYMHS's official documents, procedures, plans, reports(etc.) at your office?

- Not Applicable
- Yes
- No
- Not sure

If Yes, please indicate the documents in which it is included.

*14. Please indicate if the introduction of the Ed-LinQ Initiative has resulted in the following.

Please note that each question requires an answer.

	Yes	No	Don't Know
Inclusion of Ed-LinQ in CYMHS's policy and protocols			
Induction or training of CYMHS staff on aspects of Ed-LinQ			
New governance structures to engage schools and other partners in service collaboration			
Distribution of information on available CYMHS services to schools			
Delivering training to student welfare staff (e.g. Guidance, Nursing, et al)			
Delivering training to senior administration staff (Principal, Senior School Head et al)			
Delivering training to general teaching staff			
Defined referral processes to CYMHS or other services (e.g. headspace)			
Shared assessment/early identification of the students with mental health problems			
Case coordination/monitoring/follow up with the schools			
Case coordination/monitoring/follow up with mental health/primary health care professionals			
Provision of treatment at the school for students with mental health			
related problems:			
Introduction into schools of new social and emotional learning programs or modification			
of learning programs for all students			
Introduction into schools of new social and emotional learning programs or modification of learning programs for students with learning difficulties, conduct issues or those with a mental health problem			
Introduction into schools of new social and emotional learning programs or modification of learning programs for parents			

Other impacts? (please specify)

Child and Youth Providers of

***15. Please rate your experiences and views about the impact of the Ed-LinQ Initiative in your District / HHS on a scale from 1 - Strongly agree to 5 - Strongly disagree or indicate where you don't know.**

Please note that each question requires an answer.

	1. Strongly Agree	2. Agree	3. Neither agree nor disagree	4. Disagree	5. Strongly disagree	Don't know
The Ed-LinQ Initiative has changed the profile of referrals to CYMHS from schools.	◯	◯	◯	◯	◯	◯
The referrals of students from schools engaged in the Ed-LinQ Initiative generally results in better use of CYMHS's expertise.	◯	◯	◯	◯	◯	◯
The Ed-LinQ Initiative has increased the capacity of school staff to identify students in need of mental health services.	◯	◯	◯	◯	◯	◯
The Ed-LinQ Initiative has increased the capacity of school staff to better manage those students with sub-clinical needs at the school level.	◯	◯	◯	◯	◯	◯
The Ed-LinQ Initiative has resulted in better use of primary care services (GPs, private psychologists etc) for less acute or serious mental health problems with children and young people.	◯	◯	◯	◯	◯	◯
The Ed-LinQ Initiative has helped to build a more collaborative approach to the work of CYMHS and other service providers.	◯	◯	◯	◯	◯	◯

Child and Youth Providers of

***16. Please indicate your view on the appropriate level of resourcing that should be made available for Ed-LinQ for the next three years.**

- Resourcing to be increased substantially
- Increase resourcing with demand
- Stay about the same level
- Decrease resourcing to meet other priorities
- Decrease substantially
- Redirect resources to other programs/services

***17. Thinking about the future, please indicate below how Ed-LinQ should be changed or improved.**

***18. Overall, what is your degree of satisfaction with the Ed-LinQ Initiative?**

- Very dissatisfied
- Dissatisfied
- Neutral
- Satisfied
- Very satisfied

19. Do you have any other comments on the Ed-LinQ Initiative including any changes you would recommend?
