# Supplementary material for: Impact of Ed-LinQ: A Public Policy Strategy to Facilitate Engagement between Schools and the Mental Health Care System in Queensland, Australia
Source: Int J Environ Res Public Health. 2021 Jul 27;18(15):7924. doi: 10.3390/ijerph18157924 (PMC8345643; doi:10.3390/ijerph18157924)

GEOSPATIAL ANALYSIS OF THE ADOPTION OF THE EdLINQ PROGRAM IN QUEENSLAND (2014): Data from the Adoption Impact Ladder (AIL).

a) Adoption of the EdLINQ Program in Queensland

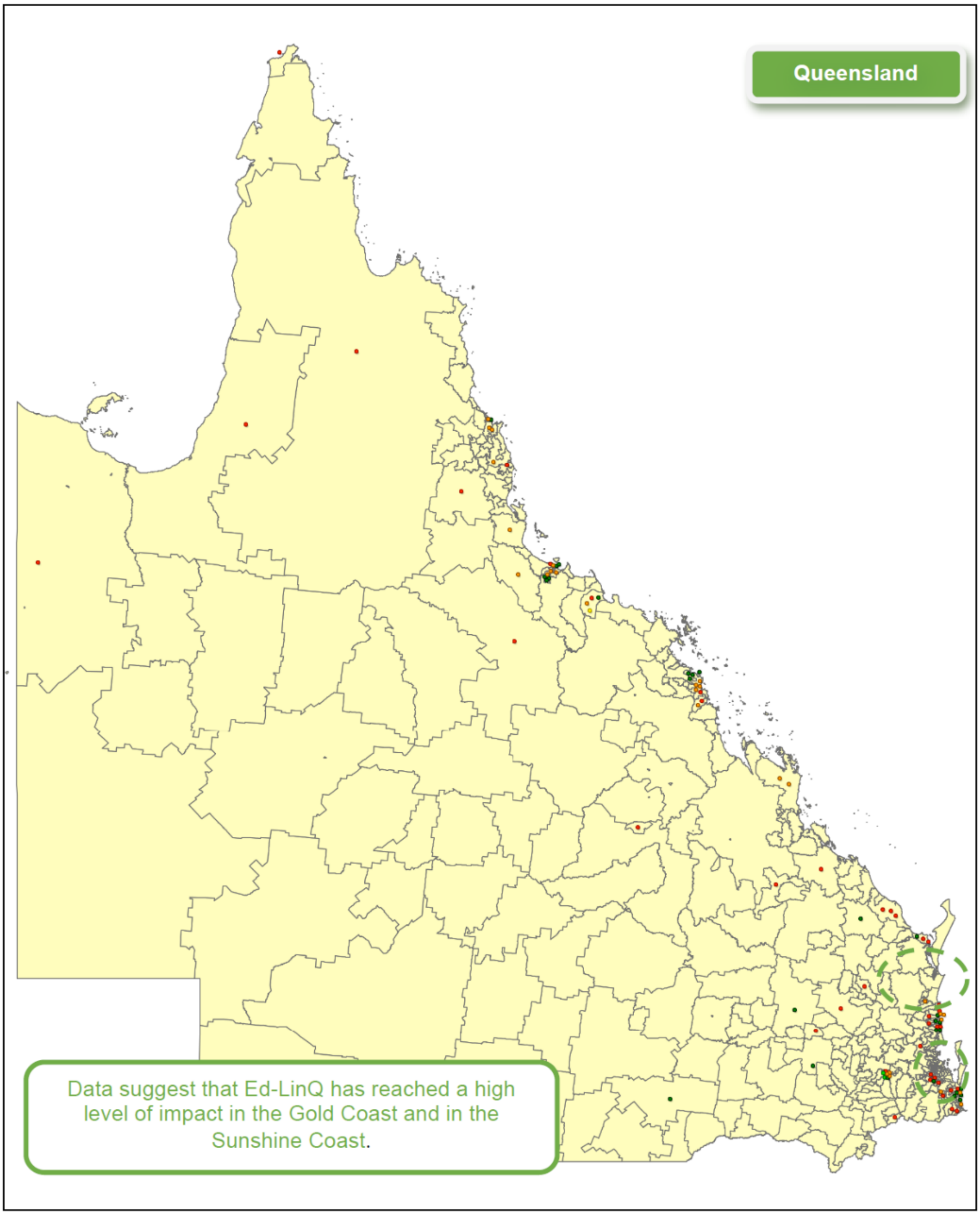

- 0. No Adoption
- 1. Awareness
- 2. Assimilation
- 4. Translation
- 5. Allocation/Provision
- 6. Routinisation

b) Adoption of the EdLINQ Program in Metropolitan Brisbane, Gold Coast and Sunshine Coast

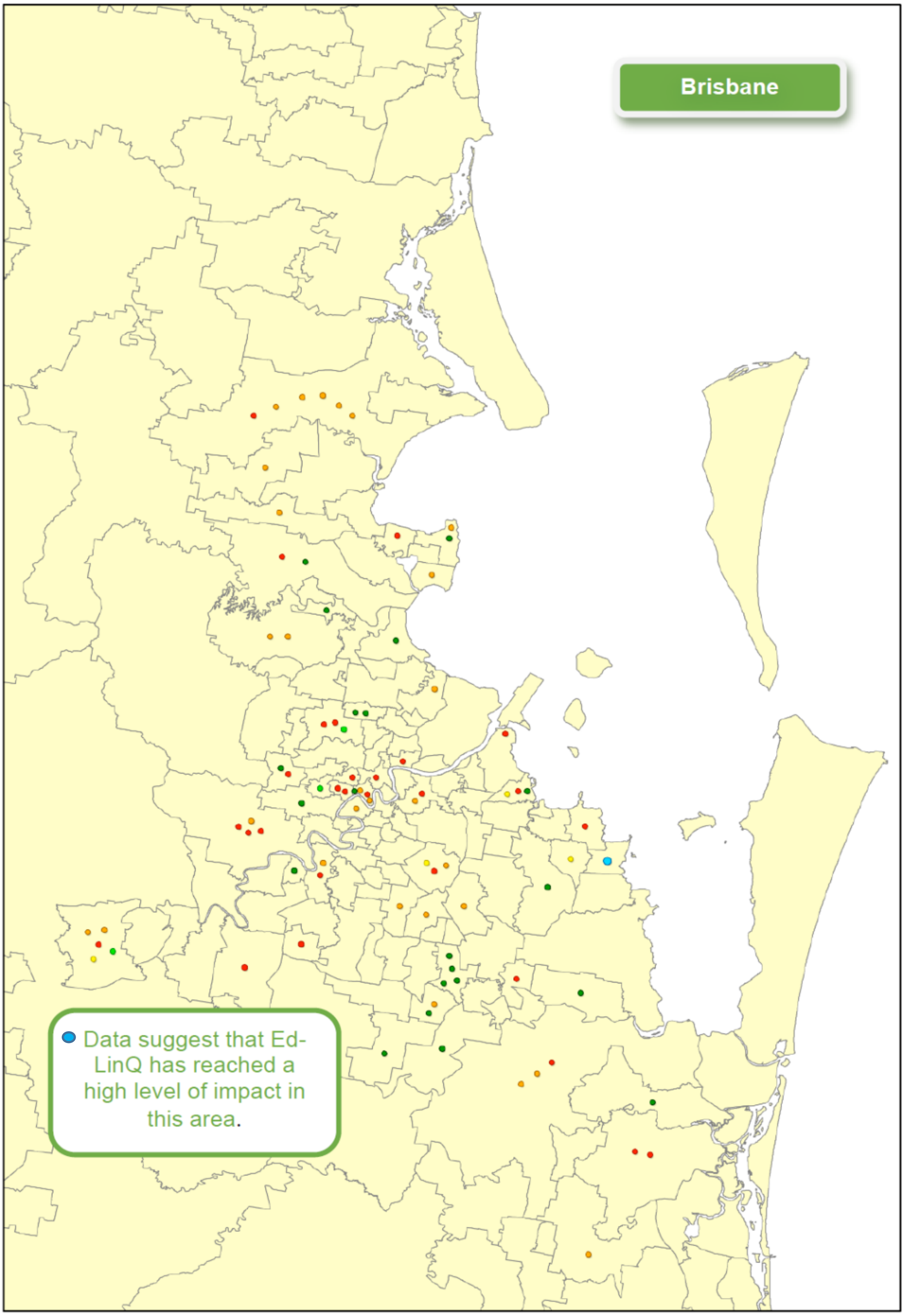

Supplement: Supplementary file 1 [file ijerph-18-07924-s001.zip › Supplementary File 4.pdf]
